# Validation of *Plasmodium falciparum* dUTPase as the target of 5′-tritylated deoxyuridine analogues with anti-malarial activity

**DOI:** 10.1186/s12936-019-3025-2

**Published:** 2019-12-03

**Authors:** Guiomar Pérez-Moreno, Paula Sánchez-Carrasco, Luis Miguel Ruiz-Pérez, Nils Gunnar Johansson, Sylke Müller, Beatriz Baragaña, Shahienaz Emma Hampton, Ian Hugh Gilbert, Marcel Kaiser, Sandipan Sarkar, Thiyagamurthy Pandurangan, Vijeesh Kumar, Dolores González-Pacanowska

**Affiliations:** 1 Instituto de Parasitología y Biomedicina López-Neyra. Consejo Superior de Investigaciones Científicas. Parque Tecnológico de Ciencias de la Salud, Avenida del Conocimiento, s/n 18016-Armilla, Granada, Spain; 2 Medivir AB, P.O. Box 1086, 14122 Huddinge, Sweden; 3 Institute of Infection, Immunity and Inflammation, College of Medical, Veterinary and Life Sciences, University of Glasgow, 120 University Place, Glasgow, G12 8TA UK; 4 Drug Discovery Unit, Division of Biological Chemistry and Drug Discovery, School of Life Sciences, University of Dundee, Dundee, DD1 5EH UK; 5 Swiss Tropical and Public Health Institute (Swiss TPH), Socinstrasse 57, 4051 Basel, Switzerland; 6 University of Basel, Petersplatz 1, 4003 Basel, Switzerland; 7 Syngene International Ltd, Biocon Park, SEZ, Bommasandra Industrial Area - Phase-IV Bommasandra-Jigani Link Road, Bangalore, 560 099 India

**Keywords:** *Plasmodium falciparum*, Deoxyuridine 5′-triphosphate nucleotido-hydrolase, 5′-tritylated deoxyuridine analogues, Mode of action

## Abstract

**Background:**

Malaria remains as a major global problem, being one of the infectious diseases that engender highest mortality across the world. Due to the appearance of resistance and the lack of an effective vaccine, the search of novel anti-malarials is required. Deoxyuridine 5′-triphosphate nucleotido-hydrolase (dUTPase) is responsible for the hydrolysis of dUTP to dUMP within the parasite and has been proposed as an essential step in pyrimidine metabolism by providing dUMP for thymidylate biosynthesis. In this work, efforts to validate dUTPase as a drug target in *Plasmodium falciparum* are reported.

**Methods:**

To investigate the role of PfdUTPase in cell survival different strategies to generate knockout mutants were used. For validation of PfdUTPase as the intracellular target of four inhibitors of the enzyme, mutants overexpressing PfdUTPase and HsdUTPase were created and the IC50 for each cell line with each compound was determined. The effect of these compounds on dUTP and dTTP levels from *P. falciparum* was measured using a DNA polymerase assay. Detailed localization studies by indirect immunofluorescence microscopy and live cell imaging were also performed using a cell line overexpressing a *Pfdut*-GFP fusion protein.

**Results:**

Different attempts of disruption of the *dut* gene of *P. falciparum* were unsuccessful while a 3′ replacement construct could recombine correctly in the locus suggesting that the enzyme is essential. The four 5′-tritylated deoxyuridine analogues described are potent inhibitors of the *P. falciparum* dUTPase and exhibit antiplasmodial activity. Overexpression of the *Plasmodium* and human enzymes conferred resistance against selective compounds, providing chemical validation of the target and confirming that indeed dUTPase inhibition is involved in anti-malarial activity. In addition, incubation with these inhibitors was associated with a depletion of the dTTP pool corroborating the central role of dUTPase in dTTP synthesis. PfdUTPase is mainly localized in the cytosol.

**Conclusion:**

These results strongly confirm the pivotal and essential role of dUTPase in pyrimidine biosynthesis of *P. falciparum* intraerythrocytic stages.

## Background

Malaria, with approximately 216 million cases each year and more than 445,000 attributed deaths reported annually, remains a devastating global health problem. The disease in humans is caused by the infection of 5 different *Plasmodium* species, among which *Plasmodium falciparum* causes most mortality, mainly in children below the age of 5 [1]. Because of the appearance of resistance to the current anti-malarial drugs and the absence of an effective vaccine, there is an urgent need for new drugs to treat the disease.

The biosynthesis of nucleotides has been highlighted as a promising pathway in the search for new anti-malarial targets, due to the high dependence of nucleotides in the intraerythrocytic stages [2]. Certain enzymes, such as dihydroorotate dehydrogenase or purine nucleoside phosphorylase, have been extensively studied as drug targets yet the potential of other steps of the pathway remains unclear [3, 4].

New anti-malarial strategies have included evaluation of the enzyme dUTPase (deoxyuridine 5′-triphosphate nucleotidohydrolase, E.C. 3.6.1.23) as a potential drug target. This enzyme is essential in both eukaryotes [5] and prokaryotes [6] and several inhibitors of the enzyme have been described that exhibit anti-malarial activity [7–10] although for certain derivatives the correlation between dUTPase inhibition and anti-malarial activity was poor. dUTPase performs a dual role by catalyzing the hydrolysis of dUTP to dUMP and PPi. It supplies the dUMP substrate for dTMP synthesis, as well as minimizes cellular levels of dUTP, avoiding misincorporation in DNA [11], which might otherwise be incorporated into DNA during replication giving rise to an activation of the base excision repair pathway and multiple cellular defects [11]. Several different oligomeric forms of the enzyme exist in nature, including monomers, dimers and trimers. *Plasmodium falciparum* and human cells contain a trimeric form of the enzyme yet selective inhibition is achievable. The trimeric dUTPases possess five highly conserved sequence motifs which participate in the active site and provide residues which are essential for activity [12]. The crystal structures of different dUTPases including the *P. falciparum* and human enzymes have been published to date [13, 14] and the molecular and structural basis for the specific inhibition of a series of triphenyl uridine derivatives has been established [13].

While dUTPase has been extensively studied with the aim of inhibitor discovery, little information exists regarding its biological role and essentiality for cell survival in *P. falciparum*. A recent study performed in *Plasmodium berghei* has suggested that dUTPase null mutants are not viable [15]. It is reasonable to assume that dUTPase is crucial to the parasite given the high AT/CG ratio (approximately 80%) in its genome and the fact that *Plasmodium* lacks dCMP/dCTP deaminase activities which would confer a central role to dUTPase in dUMP formation.

In this paper, efforts to perform gene disruption studies of the *dut* gene and to address chemical target validation of dUTPase inhibitors are described. The results suggest that the enzyme is essential for the proliferation of intraerythrocytic stage of *Plasmodium falciparum* and that dUTPase indeed is the target for certain inhibitors of the enzyme with anti-malarial activity. Detailed localization studies using different approaches have been also performed, and show that the enzyme has a ubiquitous intracellular localization appearing in cytosol, nuclei and mitochondria.

## Methods

### Chemistry

Synthesis and analytical characterization of compounds 1–4 is contained in Additional file 1.

### Plasmid design and construction

The *Pfdut* coding sequence previously isolated [13] was used as template for PCR to obtain the construct pHH1-*dut*KO. A 383 bp fragment was amplified for the KO construct, where a start codon (bold faced) at position 94 of the *Pfdut* open reading frame (motif 1) and a premature STOP codon (bold faced) at position 477 (motif 5) were introduced into the oligonucleotide. The 5′ primer AGATCT**ATG**TTTATTGTAAAAGATGA contains a BglII restriction site (underlined) and the 3′ primer CTCGAG**TCA**GGAAGTTTCATCCAGTTC a XhoI restriction site (underlined) to allow directional cloning into the previously digested transfection plasmid pHH1 (5766 bp). For the KOkon construct, a 423 bp fragment of the *Pfdut* coding sequence lacking 93 bp at the 5′ region (GCGCAGATCTATTGTAAAAGATGAAGTACTG), but retaining the 3′ terminus of the *Pfdut* gene (CGCGCTCGAG**TCA**ATATTTATTATTCGATGT) was cloned into pHH1.

The human *dut* coding sequence (497 bp) was amplified with the oligonucleotides GCGCAGATCT**ATG**CCCTGCTCTGAAGAG and GCGCGCGGCCGC**TTA**ATTCTTTCCAGTGAAACC, which introduced BglII and NotI restriction sites (underlined) that allowed the directional cloning into the expression vector pHrBl. The coding sequence for human dUTPase (HsdUTPase) cloned in the pGEM-T vector was used as template for PCR amplification. It was also used as template for the amplification and subsequent cloning of *Hsdut* pHH2, replacing the green fluorescent protein (GFP) coding sequence present in the original plasmid. The specific oligonucleotides AGATCT**ATG**CCCTGCTCTGAAGAG and CTGCAG**TTA**ATTCTTTCCAGTGG, containing the BglII and PstI restriction sites, respectively, were used for the amplification.

For the construct pHH2-*Pfdut*, the 524 bp fragment of the *Pfdut* coding sequence was amplified with the oligonucleotides AGATCT**ATG**CATTTAAAAATTGTATG and CTGCAG**TCA**ATATTTATTATTCGATG. The sense oligonucleotide contains a BglII restriction site (underlined), and the antisense oligonucleotide contains a PstI site (underlined) to allow directional cloning into the previously digested transfection plasmid pHH2 (6540 bp). The *Pfdut* gene was also amplified with the oligonucleotides AGATCT**ATG**CATTTAAAAATT and CCTAGGATATTTATTATTCGA by PCR avoiding the STOP codon and cloned in pHH2 maintaining the GFP coding sequence to obtain the construct pHH2-*Pfdut*-*GFP* that allows for expression of PfdUTPase fused to GFP in transfected *Plasmodium* parasites.

The pHH1 and pHH2 series of plasmids contain a human *DHFR* fragment mutated to encode resistance to WR99210 which allow the selection of transfected parasites while pHrBl contains the blasticidine resistance gene. All PCR products were first cloned into pGEM-T (Invitrogen), where the sequences were verified, and then subcloned into the appropriate vectors.

### *Plasmodium falciparum* cultures and transfection

*Plasmodium falciparum* asexual erythrocytic-stage parasites (3D7, a cloned line derived from isolate NF54) were cultured at 37 °C, 5% CO_2_ in 5% haematocrit using human O-positive erythrocytes and fed every day with complete culture medium (RPMI 1640 (Gibco) supplemented with 0.2% NaHCO_3_, 0.15 mM hypoxanthine, 12.5 μg/ml gentamycin (Gibco), 2% heat inactivated human serum, and 0.5% Albumax II (Gibco) [16]. Synchronized ring-stage parasites (~ 5% parasitaemia) with 5% sorbitol, were transfected with 100 μg of circular purified plasmid DNA (HiSpeed plasmid maxi kit; Qiagen) in a Genepulser from BioRad, and drug cycling commenced according to methodology described previously by Crabb et al. [17]. Following transfection, the samples were immediately mixed with 10 ml culture medium and cultured in 25-cm^2^ culture flasks until 5% parasitaemia for 48 h prior to selection with 5 nM WR99210 (Jacobus Pharmaceuticals). The parasites were maintained under drug pressure for 5 days and then the concentration of drug was decreased to 2.5 nM WR99210. The first parasites were observed after 25–28 days of selection in continuous culture. Drug cycling was performed in order to select for homologous recombination of the constructs and loss of episomal DNA. For this purpose, transfectants were cultured for 3 weeks without drug pressure followed by 4 days with WR99210 pressure. This selection cycle was repeated for several rounds, and at different stages parasite DNA was isolated and subjected to Southern blotting and PCR analyses to determine whether episomal DNA was present and if integration into the *Pfdut* gene locus had occurred.

### Southern blot analysis

Parasitized erythrocytes (5 × 10^8^ parasites per sample) were harvested by centrifugation, treated with 1.5 volumes of saponin 0.15% in PBS for 5 min at 4 °C and after adding another 5 volumes of PBS, cells were centrifuged at 5000 rpm for 10 min. Genomic DNA was isolated from parasite pellets using the QIAamp DNA Mini Kit (Qiagen). Manipulation of recombinant DNA and analysis of nucleic acids by Southern blot hybridization were carried out using standard procedures [18].

### In vitro assays

In vitro inhibitory activity against the erythrocytic stages of *P. falciparum* of overexpressing mutants was determined by using the SYBR green assay [19] and the ^3^H-hypoxanthine incorporation assay was used for IC50 determination as previously described [8]. *Plasmodium falciparum* 3D7 was cultured using standard methods, and synchronized using 5% sorbitol as previously described [20]. Compounds were dissolved in DMSO and added at different concentrations (8–500 ng/ml) to 48 h post-synchronization parasites. The content in DMSO did not exceed 0.001% to avoid solvent toxicity. Chloroquine dissolved in water was used as standard drug. Experiments were carried out at least twice independently and the different concentrations were tested in duplicate. IC50 values were calculated using a four-parameter logistic regression model using data obtained from two or three independent experiments as indicated.

### Measurement of intracellular uridine and thymidine nucleotides

The effect of different dUTPase inhibitors on dUTP and dTTP levels from *P. falciparum* cells was measured using a modified DNA polymerase assay [21]. The template sequence employed was the oligonucleotide 5′-TTTATTTATTTATTTATTTAGGCGGTGGAGGCGG-3′ and as primer sequence the oligonucleotide 5′-CCGCCTCCACCGCC-3′ was used. Saponin-isolated parasite pellets (5 × 10^8^ parasites per sample) were repeatedly washed in PBS and frozen at − 80 °C. Frozen pellets were extracted with 200 μl of cold methanol/water (1:1, v/v) vigorously by vortexing, freeze-thawed twice and centrifuged. Supernatants were collected and pellets were re-extracted with the same volume of methanol/water as previously mentioned and newly centrifuged. The combined supernatants were dried under vacuum. The residues were dissolved in 40 μl either of dUTPase buffer (34 mM Tris–HCl pH 7.8, 5 mM MgCl_2_) or dUTPase buffer plus 30 ng of HsdUTPase and incubated for 20 min at 37 °C. To stop the reaction, 60 μl of 100% methanol were added and the samples were incubated for 1 h at − 20 °C, followed by centrifugation for 20 min at 16,000×*g*. The supernatants were again dried under vacuum and the DNA polymerase assay used was modified from that of Horowitz et al. [21]. The DNA polymerase I buffer contained 34 mM Tris–HCl pH 7.8, and 50 mM MgCl_2_. After incubation with DNA polymerase I, 30 μl of each sample were incubated for 30 min at 4 °C with 470 μl of DNA polymerase I buffer containing 10% (v/v) trichloroacetic acid to precipitate the DNA. The solution was blotted onto a glass microfibre filter GF/C (Whatman) and each filter was washed under vacuum with 30 ml of a solution of 5% (v/v) trichloroacetic acid and 3 ml of ethanol, dried and the radioactivity was counted using a LS 6500 Multi-Purpose Scintillation Counter (Beckman Coulter).

### Generation of antibodies against HsdUTPase and PfdUTPase and Western blot analysis

Both proteins, PfdUTPase and HsdUTPase were purified as previously described [10]. Polyclonal antiserum against recombinant PfdUTPase and HsdUTPase was generated by immunizing rabbits with the purified protein. The monoclonal anti-PfdUTPase antibody, used for immunofluorescence analysis, was obtained as described previously [22]. Protein extracts were prepared from saponin-isolated parasites by sonication. Unless otherwise mentioned, the parasites were obtained from cultures of highly synchronized trophozoites. 10 µg of total extract of *P.* *falciparum* proteins were subjected to SDS–PAGE, blotted on an Immobilon-P membrane (Millipore) and incubated with 1:10,000 dilution of the anti-HsdUTPase antibody. Bound antibody was detected by reaction with horseradish peroxidase (HRP)-conjugate goat anti-rabbit IgG (Promega) at a dilution of 1:5000 and an ECL™ immunodetection kit (Amersham Pharmacia Biotech). Anti-Hsp70 polyclonal antibody (LifeSpan BioSciences) was used as loading control (1:10,000 dilution).

### Fluorescence microscopy

For indirect immunofluorescence microscopy, 10 ml of infected erythrocytes at 5% of parasitaemia were washed once in PBS then fixed with 4% paraformaldehyde and 0.0075% glutaraldehyde in PBS for 30 min. For visualization of the mitochondrion, cells were washed once with PBS, resuspended in the same medium containing 50 nM MitoTracker Red CMXRos (Molecular Probes), and incubated for 15 min at 37 °C before starting the fixation process. Fixed cells were washed once in PBS and then permeabilized with 0.1% Triton X-100/PBS for 10 min. Cells were washed again in PBS and then treated with 0.1 mg/ml of sodium borohydride (NaBH_4_)/PBS for 10 min to reduce any free aldehyde groups. Following another PBS wash, cells were blocked in 3% BSA/PBS for 1 h. Indirect immunofluorescence confocal microscopy using an anti-PfdUTPase monoclonal antibody or anti-HsdUTPase polyclonal antibody was performed in both wild-type cells and in overexpressing mutants. The anti-PfdUTPase monoclonal antibody (diluted 1:250) or anti-HsdUTPase polyclonal antibody (diluted 1:500) were added and incubated for 1 h. Cells were washed three times in PBS for 10 min each. Alexa Fluor goat anti-mouse 488 (Molecular Probes) diluted 1:200 or goat anti-rabbit IgG FITC-conjugate (Sigma) diluted 1:40 were added and allowed to bind for 1 h. Cells were washed three times in PBS. Cells were finally fixed with cold methanol (Merck). Samples were stained with Vectashield^®^-DAPI (Vector Laboratories) and analysed with a Leica TCS SP5 confocal microscopy system. The colocalization analysis was performed with the image processing software ImageJ.

For live cell imaging, the parasites transfected with pHH2-*Pfdut*-*GFP* were incubated at 37 °C for 15 min in medium containing 100 μg/ml of Hoechst 33342 (Invitrogen) and 50 nM of MitoTracker Red CMXRos. After washing, cells were pelleted and resuspended in 2 volumes of medium and then mounted to perform microscopy. Cells were viewed with a confocal Leica TCS SP5 microscopy system.

## Results

### The *dut* gene is essential for asexual intraerythrocytic stages of *P. falciparum*

**Fig. 1:**
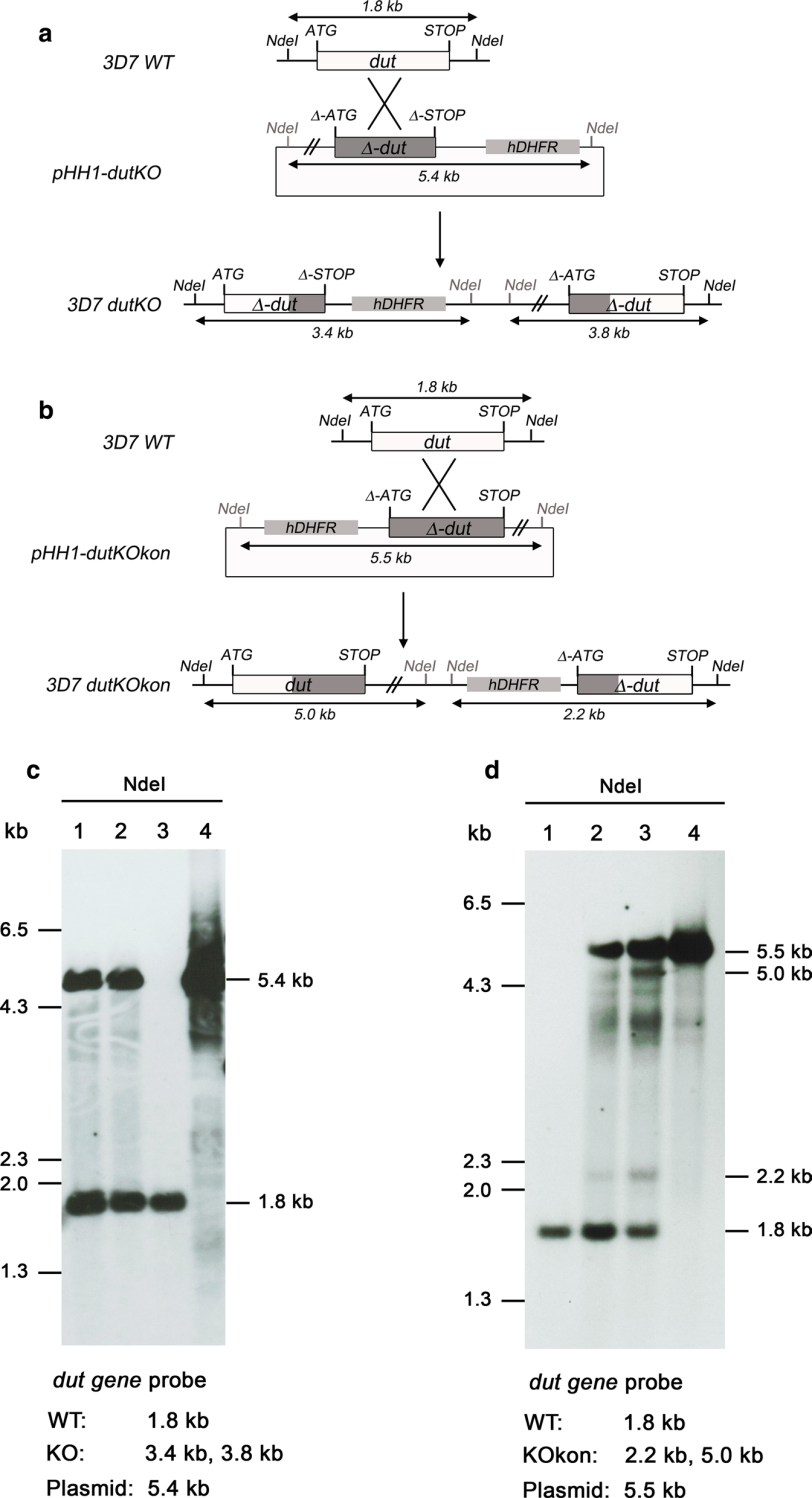
Efforts for disruption of the *Pfdut* gene by single crossover recombination. **a** After a single crossover event of the plasmid with the *dut* locus, the KO construct would lead to the generation of two truncated inactive copies of the gene. **b** The KOkon plasmid should generate a functional copy of the *dut* gene and a non-functional pseudogene upon single crossover recombination. **c** Southern blot analysis of the pHH1-dutKO transfected line after drug cycles. Genomic DNA of the cell line transfected with pHH1-dutKOk after two (lane 1) and three (lane 2) cycles of drug pressure, genomic DNA of the 3D7 parental line (lane 3) and 5 ng of purified plasmid DNA (lane 4) were digested with NdeI. The 1.8 kb band corresponding to the endogenous locus is present in the three genomic DNAs, whereas the episome (5.4 kb) is present only in lanes 1 and 2. **d** Southern blot analysis of the pHH1-dutKOkon transfected line after drug cycles. Genomic DNA of the 3D7 parental line (lane 1), the cell line transfected with pHH1-dutKOkon after two (lane 2) and three (lane 3) cycles of drug pressure and 5 ng of purified plasmid DNA (lane 4) were digested with NdeI. The 1.8 kb band corresponding to the endogenous locus is present in the three genomic DNAs, whereas the episome (5.4 kb) is present only in lanes 2 and 3. In lanes 2 and 3, two new extra bands of 2.2 kb and 5 kb were detected; the intensity of the bands increased from cycle 2 to cycle 3, corresponding to an enrichment of the culture in parasites with the plasmid integrated in the *dut* locus

To investigate the role of dUTPase in cell survival, the deletion of the gene in intraerytrocityc stages of *P. falciparum* was attempted. Different strategies were employed to replace the endogenous gene using approaches leading to single recombination as described [23, 24]. PCR fragments for single homologous recombination of the *dut* locus were obtained by PCR and cloned into the vector pHH1. Figure 1, panels a and b, indicate the strategy designed. The pHH1 knockout construct (pHH1-*dut*KO) was truncated at its 5′ end, although an ATG start codon was introduced while at the 3′ end a premature STOP codon was introduced 237 bp upstream of the natural STOP codon. This would result in the formation of two incomplete and inactive *Pfdut* copies upon single crossover recombination of the plasmid in the gene locus. In contrast, the control construct (pHH1-*dut*KOkon) retains the 3′ region of the *dut* gene but lacks the 5′ region and thus would generate upon recombination a functional copy and a non-functional pseudogene. After transfection experiments with both constructs, transfected cells were readily obtained in both cases. Transfected parasite lines were taken through several drug selection cycles in order to favour/select the population of parasites where a single crossover event in the *Pfdut* locus had occurred. Genomic DNA was isolated and analysed by diagnostic Southern blotting to establish integration events into the parasite genome. In the case of pHH1-*dut*KO, Southern blot revealed the presence of endogenous *Pfdut* (1782 bp band) and linearized plasmid (5418 bp band), but no integration events into the parasite genome were detected after several drug cycles (Fig. 1c). However in the case of transfection with pHH1-*dut*KOkon, three rounds of drug removal resulted in the integration of the construct, with two new bands of 4985 and 2217 bp as shown in Fig. 1d.

The expression of a heterologous *dut* gene in *Plasmodium* cells that may allow for subsequent removal of the endogenous copy of *Pfdut* was used as a second knockout strategy. For this purpose, the trimeric HsdUTPase was chosen. The coding sequence of human *dut* was cloned into the expression vector pHrBl to yield pHrBl-*Hsdut*. This construct was cotransfected with pHH1-*dut*KO and cells resistant to blasticidine and to the human DHFR inhibitor WR99210 were obtained. The correct expression of HsdUTPase was tested using a specific antibody (Additional file 2a) while correct transfection with pHH1-*dut*KO was verified by Southern blot (Additional file 2b). Cells efficiently overexpressed the human enzyme and showed a normal growth profile. However, after several drug cycles, no integration events were detected by Southern blotting. All together these results suggest that, although the *dut* gene is targetable, no insertions are favored which may lead to gene disruption.

### dUTPase overexpression induces resistance against inhibitors with anti-malarial activity

Four new inhibitors of plasmodial dUTPase were used. The structures of the compounds selected together with the Ki values for PfdUTPase and HsdUTPase are shown in Additional file 1 and Table 1 and are all trityl derivatives containing the uracil base. The IC50 values for intraerythrocytic stages of *P. falciparum* are also indicated. Compounds 1 and 3 contain the ribose ring while 2 and 4 are acyclic derivatives. These compounds inhibit *Plasmodium* dUTPase while are mostly inactive against the human enzyme yet exhibit anti-malarial activity in vitro at nanomolar concentrations. The most potent enzyme inhibitor against PfdUTPase was compound 3 (Ki 79 nM) while compound 1 was the least active (Ki 4.73 μM). Moreover, compounds 1 and 2 were the most active in vitro against the parasite with IC50 values of 40 and 60 nM respectively.

**Table 1:** Ki values for *Plasmodium* and human dUTPases and IC50s for intraerythrocytic stages of *Plasmodium falciparum*

	Ki PfdUTPase	Ki HsdUTPase	IC50 Pf3D7
Compound 1	4.73 ± 0.16 μM	> 100 μM	0.04 ± 0.01 μM
Compound 2	0.56 ± 0.08 μM	12.24 ± 6.91 μM	0.06 ± 0.01 μM
Compound 3	0.079 ± 0.022 μM	> 100 μM	0.12 ± 0.05 μM
Compound 4	0.61 ± 0.07 μM	> 100 μM	0.15 ± 0.03 μM

Values are the average of three independent experiments ± standard errors

**Fig. 2:**
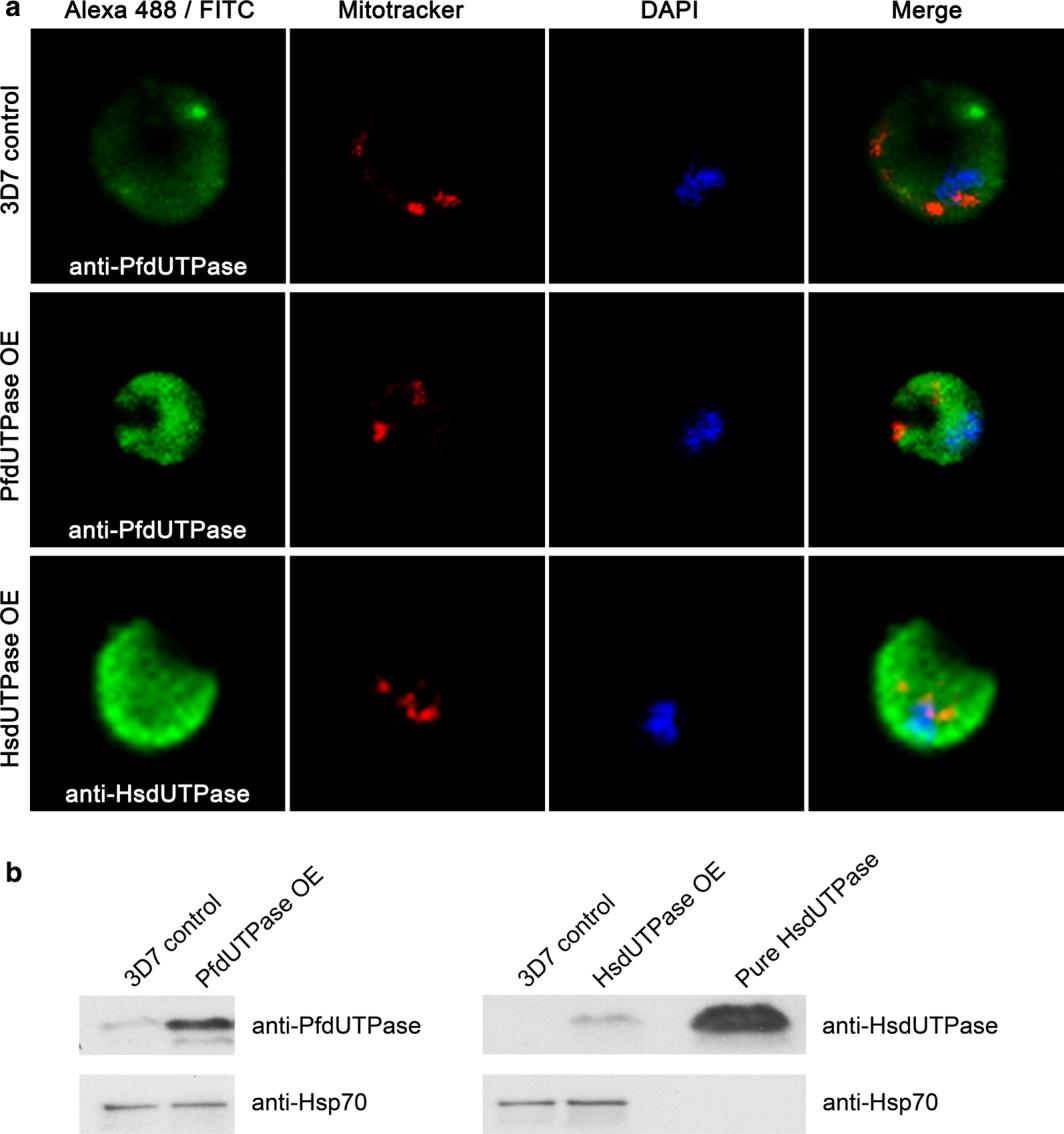
Analysis of mutants overexpressing PfdUTPase and HsdUTPase. **a** Immunofluorescence analysis of 3D7 wild-type cells (upper panels) and mutants overexpressing PfdUTPase (middle panels) and HsdUTPase (lower panels). A monoclonal antibody for PfdUTPase and a polyclonal antibody for HsdUTPase were used. Images were obtained using a confocal Leica TCS SP5 microscope and show a single optical plane. **b** Western blot analysis indicating the correct overexpression of PfdUTPase (left panel) and HsdUTPase (right panel) in parasites transfected with constructs where the coding sequence was cloned into pHH2. Western blotting was performed with polyclonal antibodies raised against PfdUTPase and HsdUTPase respectively. Hsp70 was used as loading control

Evidence that inhibitors were acting on-target in *Plasmodium* was first sought for by creating *Plasmodium* mutants overexpressing PfdUTPase and HsdUTPase. After transfection, protein overexpression in these cell lines was confirmed by Western blot and the localization within the cell by immunofluorescence. Indirect inmunofluorescence was performed using anti-PfdUTPase and anti-HsdUTPase monoclonal and polyclonal antibodies (Fig. 2a). After the quantification of Western blot signals with ImageQuant software (GE healthcare), it was established that the levels of PfdUTPase were around sixfold higher in the PfdUTPase overexpressing line while HsdUTPase although detectable, overexpressed to a lesser degree (Fig. 2b).

**Fig. 3:**
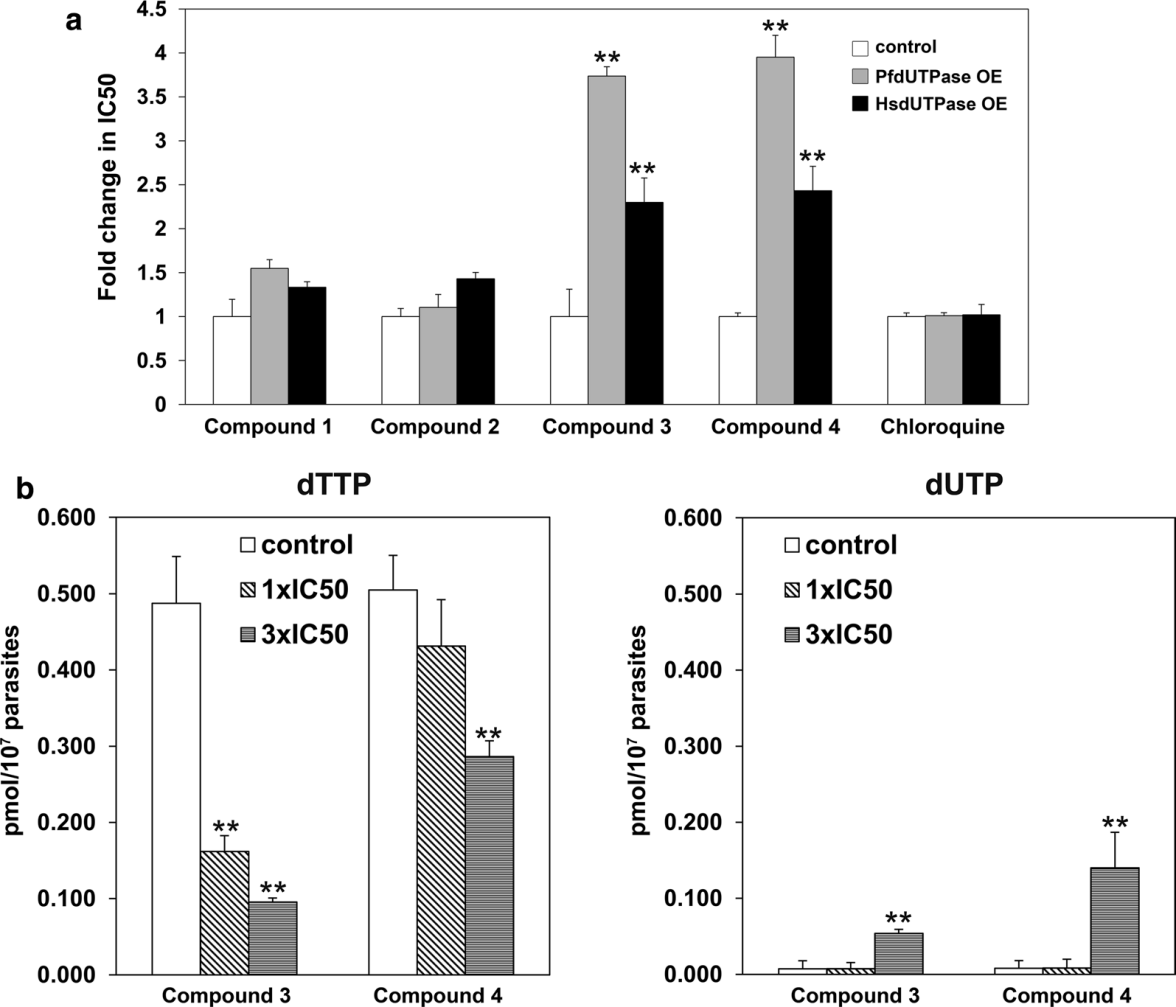
Effect of dUTPase inhibitors on cell growth and nucleotide pools. **a** Resistance induced in *Plasmodium* cultures by overexpression of different dUTPases indicated by the fold change observed in the value of IC50 obtained for transfected *Plasmodium falciparum* lines overexpressing PfdUTPase (PfdUTPase OE) and human dUTPase (HsdUTPase OE) with regard to the parental line 3D7. Chloroquine was used as reference compound. **b** Measurements of the dTTP (left panel) and dUTP pools (right panel) after incubation for 20 h with compounds 3 and 4 at different concentrations (IC50 and threefold the IC50). Determinations of IC50 values and nucleotide levels were performed in triplicate and the experiments were performed twice. Average values of both experiments are indicated and error bars represent the standard error. The asterisks show significant differences calculated by the Student’s t-test. **p < 0.01

Subsequently, the IC50 for each cell line with each compound and the fold change relative to the 3D7 IC50 was calculated (Fig. 3a). For compound 3, one of the most potent dUTPase inhibitors with a Ki of 79 nM, overexpression resulted in a 3.8-fold reduction in potency in the case of PfdUTPase (Fig. 3a) and a 2.3 reduction when overexpressing HsdUTPase. In the case of compound 4, IC50 values were increased 4 and 2.5-fold in the PfdUTPase and HsdUTPase overexpressing lines respectively. However, IC50s for compounds 1 and 2, exhibited minor changes with values ranging between 1.55 and 1.10, suggesting that dUTPase is probably not the major target responsible for the anti-malarial effect of these latter analogues. The IC50 for chloroquine as a reference compound was also determined in overexpressing mutants. No significant effects were denoted indicating that changes in compound susceptibility are specific to dUTPase inhibitors.

### Incubation with dUTPase inhibitors results in decreased dTTP and increased dUTP levels

Perturbation of the production of dUMP for dTTP biosynthesis by different dUTPase inhibitors was monitored by determining dTTP levels in parasites. Considering the central role of dUTPase in the production of dUMP for dTMP synthesis via thymidylate synthase-dihydrofolate reductase, the incubation of *P. falciparum* with dUTPase inhibitors should produce a decrease in dTTP and an increase in dUTP pools in treated cells thus resulting in an imbalance in the dUTP/dTTP ratio.

The levels of intracellular dTTP and dUTP were measured using the DNA polymerase based assay in *Plasmodium* cultures incubated with compounds 3 and 4. Parasites for dUTP and dTTP measurements were obtained from highly synchronized cultures in schizont stage after 20 h of treatment with concentrations of compound that corresponded with 1× and 3× the IC50 concentration. As shown in Fig. 3b both compounds induced a significant decrease in dTTP and an increase in the dUTP pool at high concentrations further supporting that inhibition of dUTPase has an impact on dNTP pools and subsequently on parasite replication.

### PfdUTPase is mainly localized in the cytosol and expressed in a stage-dependent manner

Indirect immunofluorescence (Fig. 2a) allowed for quantification of the intracellular distribution of the enzyme. In trophozoites the signal can be associated mostly with the cytosol although a certain overlap occurs with both mitochondria and nuclei. Intracellular localization of dUTPase in mutants overexpressing PfdUTPase (Fig. 2a) was also analysed and appeared to be similar to what was observed in the parental cell line.

**Fig. 4:**
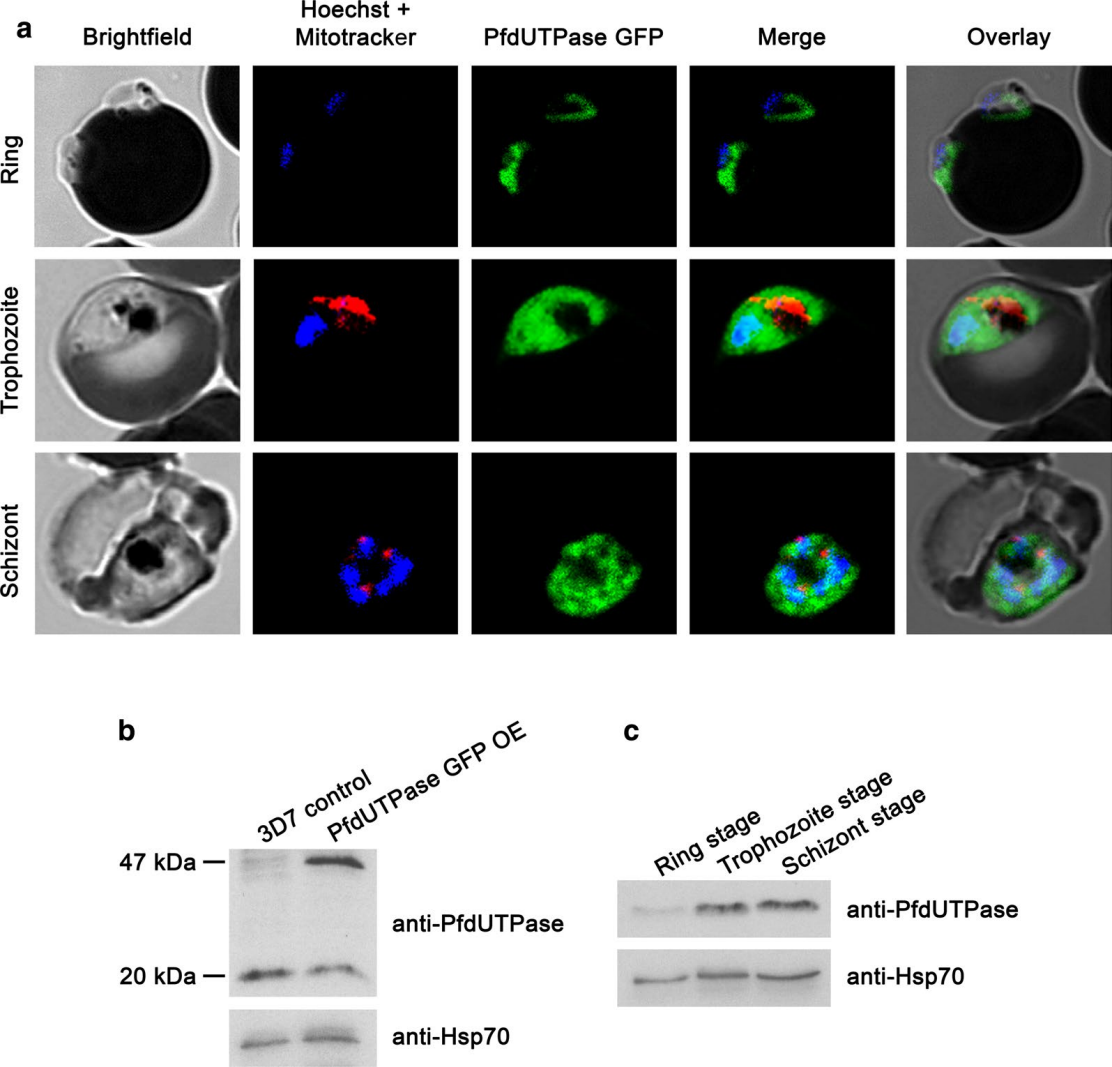
Expression and intracellular localization of dUTPase at different stages of the intraerythrocytic cycle. The intracellular localization of dUTPase was assessed by live cell fluorescence microscopy of cells overexpressing PfdUTPase fused to GFP. **a** In vivo microscopy of parasites transfected with the plasmid pHH2-*Pfdut*-*GFP*. Cells were previously stained with Hoechst33342 and Mitotracker for nucleus and mitochondria localization. Images were taken with a confocal Leica TCS SP5 microscopy system and show a single optical plane. **b** Western blotting of extracts of parasites overexpressing PfdUTPase-GFP using a polyclonal anti-PfdUTPase antibody. A band of approximately 46.5 kDa appears only in transfected parasites (PfdUTPase GFP OE), corresponding to the fusion protein dUTPase-GFP. **c** Expression of PfdUTPase in 3D7 wild-type cells throughout the intraerythrocytic cycle. Protein extracts of parasites in ring (R), trophozoite (T) and schizont (S) stages were subjected to Western blotting with the polyclonal dUTPase antibody. Anti-Hsp70 was used as loading control

Additionally, analysis was performed with a cell line overexpressing a *Pfdut*-GFP fusion protein and live cell fluorescence microscopy allowing for direct observation of dUTPase. In this case MitoTracker Red was used for mitochondria visualization and Hoechst33342 for nuclei. As shown in Fig. 4a, a similar protein distribution to the one observed using indirect immunofluorescence was obtained. The correct expression of the fusion protein was confirmed by Western blot with polyclonal anti-PfdUTPase antibody (Fig. 4b). A single band of approximately 20 kDa was detected in the 3D7 parental line, while two bands, one corresponding to the native PfdUTPase and a second of approximately 47 kDa corresponding to the PfdUTPase-GFP fusion protein, could be detected in pHH2-*Pfdut*-*GFP* transfected parasites.

Finally, Western blotting allowed for quantification with ImageQuant software (GE healthcare) during the life cycle using extracts of highly synchronized 3D7 wild-type parasites and showed that PfdUTPase levels in trophozoites and schizonts (3D7 cells) were 3.3 and 3.8 times respectively those observed in ring forms (Fig. 4c).

## Discussion

Nucleotide metabolism has been highlighted as a source of enzymes for target‐based drug development in *Plasmodium* [25]. *Plasmodium falciparum* dUTPase had been extensively studied in the search of potent inhibitors showing anti-malarial properties [7, 8, 10] yet, while presumed to be indispensable for growth considering its central role in providing dUMP for thymidylate biosynthesis, its essential character has not been demonstrated. Trimeric dUTPases have been shown to be essential for viability in several organisms, such as *Saccharomyces cerevisiae* [5], *Escherichia coli* [6], or *Mycobacterium smegmatis* [26]. Likewise, knockout mutants for the dimeric enzymes present a growth defective phenotype [27]. The loss of viability has been associated with an imbalance in the dUTP/dTTP ratio. Thus in the absence of dUTPase an increase in this ratio results in massive incorporation of uracil during replication due to an expansion of the dUTP pool. Indeed *Saccharomyces cerevisiae* [5] and *Trypanosoma brucei* [28] knockout mutants are thymidine auxotrophs. Human and *Plasmodium* dUTPases share a similar overall fold yet selective inhibition has been shown to be feasible. Thus, a series of trityl and deoxyuridine derivatives and their acyclic analogues can inhibit *P. falciparum* dUTPase and show anti-malarial activity [8]. Structural data obtained for enzyme–inhibitor complexes evidenced that the triphenylmethane group of these compounds interacts with the side chains of residues Phe46 and Ile117 that are part of a hydrophobic pocket present in *Plasmodium* dUTPase different from the phosphate binding site [13]. These residues are replaced by Val42 and Gly87 in the human enzyme [13]. The present study provides genetic evidence suggesting that PfdUTPase is indeed indispensable for erythrocytic stages of *P. falciparum*. The *dut* locus could not be disrupted, yet could be correctly targeted. The inability to knockout the gene using a simple crossover strategy has been understood as evidence that supports the essentiality of the gene [29]. Alternative strategies such as the complementation by HsdUTPase in a KO background were not feasible. Several reasons may explain this observation. Thus, it is possible that levels of human enzyme are inadequate to sustain dTMP biosynthesis. In addition, the existence of protein–protein interactions specific to PfdUTPase and that are essential for parasite viability cannot be discarded. In support of the essential character of *Pfdut*, a recent study using transposon mutagenesis has defined the mutability and fitness costs for over 87% of *P. falciparum* genes and established 2680 genes as essential for optimal growth of asexual blood stages in vitro [30]. The coding sequence for PfdUTPase appeared in this study as non-mutable. Since the absence of insertions in the CDS was considered as an indicator that disruptions are lethal, the data is also indicative of *Pfdut* being essential [30]. In addition, the enzyme appears to be essential in *P. berghei* since deletion of dUTPase failed after several attempts suggesting a crucial role during intraerythrocytic development [15].

While multiple studies have shown that PfdUTPase can be efficiently inhibited in vitro and that enzyme inhibitors also exhibit antiplasmodial activity, no study has been performed in order to validate that indeed the intracellular target of these compounds is dUTPase. Most inhibitors discovered to date are uracil-based compounds that interact with the substrate binding site. Specifically, 5′-tritylated nucleosides are selective inhibitors of the *P. falciparum* enzyme versus the HsdUTPase [31]. Further modifications of 5′-tritylated deoxyuridine derivatives gave rise to a generation of acyclic analogues that showed a good correlation between enzyme inhibition and antiparasitic activity [8, 10].

For chemical validation, different compounds that exhibit inhibition of both PfdUTPase and antiplasmodial activity were chosen. Mutants overexpressing PfdUTPase or HsdUTPase are expected to confer resistance if the enzyme is the primary target. When comparing the fold change in IC50 of the different compounds tested, the action of compounds 3 and 4 was clearly dependent on enzyme levels. Compound 3 is a 3′carbamate and a potent inhibitor of *Plasmodium* dUTPase while compound 4 is an acyclic 3′ urea that appears to be eightfold less active against the enzyme than compound 3 although both share the characteristic of exhibiting a bulky carboxybenzene substituent in the 3′ position. Both exhibit significant antiplasmodial activity in vitro and are selective versus the human enzyme. The lower ability of HsdUTPase to counteract the effect of the inhibitor, although still doubling the original IC50, can be due to low protein levels or a reduced ability of HsdUTPase to substitute the *Plasmodium* enzyme. While not performed in the present study, overexpression of a catalytic mutant would not confer resistance to the inhibitors thus reinforcing the concept that dUTPase is the target of compounds 3 and 4.

In the case of compounds 1 and 2 (3′ urea derivatives) dUTPase inhibition does not appear to relate to the antiplasmodial activity. Indeed, the Ki values for PfdUTPase for compounds 1 and 2 are respectively nearly two and one orders of magnitude higher than their anti-malarial activity in vitro pointing towards the existence of other intracellular targets. Hence, while certain compounds clearly involve inhibition of dUTPase as their main target within the cells, for others additional modes of action should be invoked, although these remain to be established. The pronounced decrease in dTTP and increase in dUTP in treated *Plasmodium* cultures further reinforces the idea that compounds 3 and 4 are acting through inhibition of dUTPase. Depletion of nucleotide pools upon incubation with specific inhibitors also underscores not only the importance of dUTPase in keeping low levels of dUTP, but also its key role in providing dUMP for dTTP biosynthesis (Additional file 3).

## Conclusion

In summary, here evidence is provided that dUTPase is a valuable target to be considered for target-based drug design. The information presented will contribute to the design of potent PfdUTPase inhibitors with anti-malarial activity. The future challenge resides in the identification of selective, stable drug-like compounds with potent activity that may present features that allow for their use in vivo.

## Supplementary information

**Additional file 1.** Identification of compounds. ^1^H-NMR spectra of compounds 1–4.

**Additional file 2.** Attempt to disrupt the *Pfdut* gene in *Plasmodium falciparum* overexpressing HsdUTPase. **a** Overexpression of HsdUTPase in *Plasmodium* 3D7 cells cotransfected with pHrBl-*Hsdut* and pHH1-*dut*KO. Western blotting was performed with polyclonal antibodies raised against PfdUTPase and HsdUTPase. Hsp70 was used as loading control. Lane 1, extracts of non-transfected 3D7 cells; lane 2, extracts of cells cotransfected with pHrBl-*Hsdut* and pHH1-*dut*KO. **b** Southern blot analysis of the cell line cotransfected with pHrBl-*Hsdut* and pHH1-*dut*KO after one (lane 2), two (lane 3) and four (lane 4) cycles of drug pressure. Genomic DNA of the 3D7 parental line (lane 1) and the transfected cell line were digested with NdeI. The Southern blot was probed with a fragment of the *Pfdut* coding sequence. The 1.8 kb band corresponding to the endogenous locus is present in the four genomic DNAs, whereas the episome (5.4 kb) is present only in lanes 2, 3 and 4. Extra bands of 3.4 kb and 3.8 kb indicative of integration events were not detected.

**Additional file 3.** Scheme depicting the role of dUTPase in pyrimidine metabolism. UMP/CMPK (putative), uridine monophosphate/cytidine monophosphate kinase; NDK, nucleoside-diphosphate kinase; CTPS, cytidine triphosphate synthetase; RNR, ribonucleotide reductase; dUTPase, deoxyuridine 5’-triphosphate nucleotido-hydrolase; DHFR-TS, dihydrofolate reductase / thymidylate synthase; TMPK, thymidine monophosphate kinase.

## Data Availability

Not applicable.

## Abbreviations

dUTPase: deoxyuridine 5′-triphosphate nucleotido-hydrolase
*Pfdut*: *Plasmodium falciparum* dUTPase coding sequence
*Hsdut*: human dUTPase coding sequence
dNTP: deoxynucleoside triphosphate
KO: knockout
KOkon: control knockout
GFP: green fluorescent protein
DHFR: dihydrofolate reductase
Hsp70: heat shock protein 70
DMSO: dimethyl sulfoxide
PBS: phosphate buffered saline
CDS: coding DNA sequence
