# Supplementary material for: Validation of Plasmodium falciparum dUTPase as the target of 5′-tritylated deoxyuridine analogues with anti-malarial activity
Source: Malar J. 2019 Dec 3;18:392. doi: 10.1186/s12936-019-3025-2 (PMC6889535; doi:10.1186/s12936-019-3025-2)
Supplement: Supplementary file 1 — Additional file 1. Identification of compounds. 1H-NMR spectra of compounds 1–4. [file 12936_2019_3025_MOESM1_ESM.docx]

**Synthesis and analytical characterisation of compounds 1-4.**

**General**

Normal phase TLC was carried out on pre-coated silica plates (Kieselgel 60 F_254_, BDH) with visualisation *via* U.V. light and/or ninhydrin solution. ^1^H-NMR spectra were recorded at a Bruker Avance DPX500 spectrometer 300 MHz and 500 MHz spectrometers using te applied solvent simultaneously as internal standard. Chemical shifts (δ) are given in ppm together with the relative frequency, assignment, the coupling constants (*J*, Hz) and the multiplicity: singlet (s), broad singlet (bs), doublet (d), triplet (t), quartet, quintuplet, sextet, septuplet, multiplet (m), broad multiplet (bm). ^13^C-NMR spectra were recorded at a Bruker 500 MHz NMR spectrometer using the applied solvent simultaneously as internal standard. 2D NMR spectra COSY (*H-H*) was acquired. Chemical shifts (δ) are given in ppm. In some cases, the numbering and the nomenclature system used for the structures are not referring to IUPAC nomenclature in order to aid the NMR interpretation. High resolution mass spectra were performed respectively on a Waters ZQ4000 and a Finningan MAT 95XP at EPSRC National Mass Spectrometry Service centre in the Chemistry department, University of Wales Swansea, Swansea, Wales, UK. Flash chromatography was performed using Combiflash Companion and prepacked column (silica gel and C18 reverse phase) purchased from Redisep (Presearch) or Sylicycle (Anachem). Preparative HPLC was performed using a Gilson (321-Pump, 153-UVVis Detector) equipped with a Gilson liquid handler for injection and fraction collection and XBridge Prep C18, 5μm, ODB, 19x100mm column (Waters) with 0.1% ammonia in water (solvent A) and acetonitrile (solvent B) as mobile phase. Solvents and reagents were purchased from chemical companies and used without further purification. Dry solvents were purchased in sure sealed bottles stored over molecular sieves.

**Abbreviations**

| δ | chemical shift |
| --- | --- |
| d | Doublet |
| *dd* | double doublet |
| DCM | Dichloromethane |
| DMF | Dimethylformamide |
| ES | low resolution electro spray mass spectroscopy |
| EtOAc | Ethyl acetate |
| HPLC | high performance liquid chromatography |
| HRMS | high resolution mass spectrum |
| LCMS | liquid chromatography mass spectrometry |
| m | Multiplet |
| min | Minutes |
| m/z | mass spectrum peak |
| MeOH | Methanol |
| Ms | Mesyl |
| NaN_3_ | Sodium azide |
| NMR | nuclear magnetic resonance |
| Pd/C | Palladium on carbon |
| q | Quartet |
| rt | room temperature |
| s | Singlet |
| t | Triplet |
| THF | Tetrahydrofuran |
| TLC | thin layer chromatography |

**Methods**

Compound **1**, a 5’-Tritylated-3’-amino nucleoside, with xylo configuration at the 3’-position, was synthesised in four steps. Starting from a previously described 5’-tritylamino deoxyuridine analogue, the configuration at 5’ position was inverted by the generation of a 3’-mesylate and displacement with sodium azide. Hydrogenation of the azide in the presence of catalytic Pd/C under atmospheric pressure in methanol followed by reaction of the amine with ethyl isocyanatopropionate in THF gave rise to compound **1** (**Scheme 1**).

**Scheme 1:** (i) MsCl, pyridine, rt, yield: 90%; (ii) NaN_3_, DMF, 150^0^C, yield: 56%; (iii) Pd/C, H_2_, MeOH, rt, yield: 35%; (iv) ethyl isocyanatopropionate, THF, 40^0^C.

**5’-tritylamino-3’-mesyloxy-2’-deoxyuridine**

To a solution of 5’-tritylamino-2’-deoxyuridine [1] (30 g, 63.8 mmol) in pyridine (300 mL) cooled to 0^o^C was added methanesulfonylchloride (17.3 mL, 223.6 mmol). The solution was allowed to reach room temperature and stirred for 4 h. After completion of the reaction, the reaction mixture was poured into ice-water and extracted with ethyl acetate (3 x 200 mL). The combined organics was washed with 0.5N HCl (100 mL) followed by brine (100 mL) and dried over sodium sulfate. The solvent was removed under reduced pressure and the titled compound was obtained and taken to the next step without further purification (31 g, yield 90%). ^1^H NMR (400 MHz, DMSO-*d*_6_): *δ* 11.41 (bs, 1H),7.63 (d, 1H, *J* = 8.4 Hz), 7.40-7.21 (m, 15 H), 6.14 (t, 1H, *J* = 6.8 Hz), 5.46 (d, 1H, *J* = 8.4 Hz), 5.31-5.30 (m, 1H), 4.18-4.17 (m, 1H), 3.43-3.38 (m, 1H), 3.29-3.26 (m, 1H), 3.24 (s, 3H), 2.56-2.51 (m, 2H), 1.09 (t, 1H, *J* = 6.8 Hz); LC/MS (ESI/APCI) *m*/*z* for C_29_H_29_N_3_O_6_S calcd 547.18; found 582.0 (M+Cl).

**3-azido-5-tritylamino-2,3-dideoxy-β-D-xylofuranosyluracil**

To a mixture of sodium azide (8.4 g) in DMF (100 mL), a solution of 5’-tritylamino-3’-mesyloxy-2’-deoxyuridine(20 g) in DMF (100 mL) was added dropwise over a period of 5 min. Then the mixture was heated at 150^o^C for 30 min. Solvents were removed under reduced pressure. The residue was dissolved in CHCl_3_ (200 mL) followed by filtration. The filtrate was concentrated followed by purification through flash column chromatography on silica gel to obtain the title compound as an off-white solid (10 g, 56% yield).^1^H NMR (400 MHz, DMSO-*d*_6_): *δ* 11.31 (bs, 1H),7.44-7.17 (m, 16H), 5.99 (t, 1H, *J* = 5.6 Hz), 5.59 (d, 1H, *J* = 8 Hz), 4.53-4.51 (m, 1H), 4.13-4.12 (m, 1H), 2.89 (t, 1H, *J* = 5.2 Hz), 2.77-2.71 (m, 1H), 2.35-2.27 (m, 2H), 2.09-2.07 (m, 1H); LC/MSD-Trap *m*/*z* for C_28_H_26_N_6_O_3_ calcd 494.21; found 493.10 (M-1) & 529.0 (M+Cl).

**3-amino-5-tritylamino-2,3-dideoxy-β-D-xylofuranosyluracil**

A solution of 3-azido-5-tritylamino-2,3-dideoxy-β-D-xylofuranosyluracil(6 g) in MeOH (30 mL) was added 10% Pd/C (500 mg) and hydrogenated overnight under normal atmospheric pressure. After filtration through celite bed the filtrate was concentrated and the crude was purified by flash column chromatography on silica gel (4% MeOH in CHCl_3_) to afford the titled compound as an off white solid (3 g, yield 53%) ^1^H NMR (400 MHz, DMSO-*d*_6_): *δ* 11.21 (bs, 1H),8.01 (d, 1H, *J* = 8 Hz), 7.43-7.18 (m, 15H), 5.97 (t, 1H, *J* = 5.6 Hz), 5.48 (d, 1H, *J* = 8 Hz), 3.98-3.96 (m, 1H), 3.54-3.53 (m, 1H), 2.91 (t, 1H, *J* = 7.6 Hz), 2.38-2.29 (m, 2H), 1.72-1.69 (m, 1H); LC/MSD-Trap *m*/*z* for C_28_H_28_N_4_O_3_ calcd 468.22; found 467.30 (M-1) & 503.1 (M+Cl).

**3- *N*-(4-(carboethoxy)butylurea)-5-tritylamino-2,3-dideoxy-β-D-xylofuranosyluracil (Compound 1)**

3-amino-5-tritylamino-2,3-dideoxy-β-D-xylofuranosyluracil (150 mg) was dissolved in anhydrous THF (4.5 ml) and ethyl isocyanatopropionate (51 μl) was added. The reaction was heated at 40°C for 2h. The precipitate was filtered, washed with ethyl ether and dissolved in methanol. After removing solvents the title compound was obtained as white solid. ¹H-NMR (500 MHz, d-6 DMSO) **** 11.28 - 11.28 (s, 1H), 7.44 - 7.38 (m, 7H), 7.30-7.27 (m, 6H), 7.20-7.17 (m, 3H), 5.84 - 5.77 (m, 3H), 5.56 (d, 1H, *J* = 8.1 Hz), 4.31 (dd, 1H, *J* =3.6, 7.2 Hz), 4.13 (dd, 1H, *J* = 6.1, 11.3 Hz), 4.05 (q, 2H, *J* = 7.1 Hz), 3.01 - 2.85 (m, 3H), 2.73 - 2.64 (m, 1H), 2.30 - 2.24 (m, 1H), 2.24 - 2.19 (m, 2H), 2.16 - 2.09 (m, 1H), 1.79 - 1.73 (m, 1H), 1.59 - 1.51 (m, 2H), 1.18 (t, 3H, *J* = 7.1 Hz) ppm. ^13^C-NMR (125 MHz, d-6 DMSO)  173.1, 163.7, 158.2, 150.9, 146.4, 140.7, 128.8, 128.2, 126.6, 102.1, 84.2, 82.6, 70.8, 60.2, 49.9, 43.2, 39.1, 38.4, 31.5, 25.9, 14.6 ppm. LC-MS(ES+) m/z 626.3 (M+H)^+^. HRMS (ES+) for C_35_H_40_N_5_O_6_^+^(M+H)^+^  calcd 626.2979, found 626.3034.

Compound **3**, a 3’-carbamate derivative of deoxyuridine, was prepared from 5-tritylamino-2’,5’-dideoxyuridine with ethyl isocyanate benzoate in the presence of a sodium hydride in DMF followed by hydrolysis of the ester with 10 eq of lithium hydroxide (LiOH) in a mixture 1/1 of dioxane/water (**Scheme 2**).

**Scheme 2:** (i) ethyl isocyanatobenzoate, NaH, DMF, rt, yield: 33%; (ii) LiOH, dioxane/water, rt, yield: 18%.

**3’-*O*-(4-(carboethoxy)phenylurethane)-5’-*N*-tritylamino-2’,5’-dideoxyuridine**

To a solution of 5-tritylamino-2’,5’-dideoxyuridine[1] (0.155 g, 0.33 mmol) and ethyl isocyanatobenzoate (69 mg, 0.363 mmol) in anhydrous DMF (9 ml), NaH (60% in mineral oil, 66 mg, 1.65 mmol) was added and the mixture was stirred at room overnight. The reaction was quenched with water (5 ml) and extracted with ethyl acetate (2x30 ml). The combined organic layers were dried over magnesium sulphate and the solvents were removed under vacuum. The product was purified by column chromatography using a 4g disposable silica cartridge using A (DCM) and B (5% MeOH in DCM) and the following gradient: 2 min hold 100% A, 25 min ramp to 100% B and 1 min hold at 100%B. The desired product was dissolved in acetonitrile (7.5 ml) and further purified by preparative HPLC to obtain the title compound as a white solid (72 mg, 33% yield). ^1^H-NMR (500 MHz, CDCl_3_)** ** 9.59 (bs, 1H), 8.04 (d, 2H, *J*=8.7 Hz), 7.66 (bs, 1H), 7.52-7.49(m, 8H), 7.31-7.28(m, 6H), 7.22-7.17 (m, 4H), 6.31 (dd, 1H, *J*=6.2, 7.5 Hz), 5.70 (d, 1H, *J*=8.1 Hz), 5.25-5.24 (m, 1H), 4.39 (q, 2H, *J*=7.1 Hz), 4.27-4.24 (m, 1H), 2.72-2.69 (m, 1H), 2.49-2.45 (m, 1H), 2.36 (dd, 1H, *J*=7.1, 12.2 Hz), 2.15 (bs, 1H, NH), 2.06 (td, 1H, *J*=7.0, 14.1 Hz), 1.41 (t, 3H, *J*=7.1 Hz) ppm. ^13^C-NMR (125 MHz, CDCl_3_)  166.2 (C), 163.1 (C), 152.2 (C), 150.5 (C), 145.4 (C),  (C),  (CH),  (CH),  (CH), 128.0 (CH), 126.6 (CH), 125.6 (C), 117.8 (CH), 103.2 (CH), 84.4 (CH), 84.2 (CH), 75.3 (CH), 70.7 (C), 60.9 (CH_2_), 45.9 (CH_2_), 37.4 (CH_2_), 14.4 (CH_3_) ppm. LC-MS(ES+) m/z 661.3 (M+H)^+^. HRMS (ES+) for C_38_H_37_N_4_O_7_ (M+H)^+^  calcd 661.2657, found 661.2649.

**3’-*O*-(4-(carboxy)phenylurethane)-5’-*N*-tritylamino-2’,5’-dideoxyuridine (Compound 3)**

3’-*O*-(4-(carboethoxy)phenylurethane)-5’-*N*-tritylamino-2’,5’-dideoxyuridine(46 mg, 0.069 mmol) was dissolved in dioxane/water 4/1 (5 ml) and lithium hydroxide (17 mg, 0.69 mmol) was added. The reaction was stirred at room temperature overnight. The reaction mixture was partitioned between ethyl acetate (50 ml) and water (10 ml). The aqueous layer was washed with ethyl acetate (50 ml) and then the pH was adjusted to 4 with 2% HCl. The product was extracted with ethyl acetate (2x50 ml), the organic layers were combined, dried over magnesium sulphate and the solvents were removed under reduced pressure. The residue was dissolved in acetonitrile (4 ml) and purified by preparative HPLC to obtain the title compound as a white solid (8 mg, 18% yield). ^1^H-NMR (500 MHz, CDCl_3_)  9.59 (bs, 1H), 8.04 (d, 2H, *J*=8.7 Hz), 7.66 (bs, 1H), 7.52-7.49(m, 8H), 7.31-7.28(m, 6H), 7.22-7.17 (m, 4H), 6.31 (dd, 1H, *J*=6.2, 7.5 Hz), 5.70 (d, 1H, *J*=8.1 Hz), 5.25-5.24 (m, 1H), 4.39 (q, 2H, *J*=7.1 Hz), 4.27-4.24 (m, 1H), 2.72-2.69 (m, 1H), 2.49-2.45 (m, 1H), 2.36 (dd, 1H, *J*=7.1, 12.2 Hz), 2.15 (bs, 1H), 2.06 (td, 1H, *J*=7.0, 14.1 Hz), 1.41 (t, 3H, *J*=7.1 Hz) ppm. ^13^C-NMR (125 MHz, d-4 MeOD) ** ** 166.0 (C), 154.6 (C), 152.0 (C), 147.2** **(C), 143.6 (C), 142.2(CH), 131.7** **(CH), 129.9(CH), 128.9 (CH), 127.5 (CH), 118.7 (CH), 103.1 (CH), 86.4 (CH), 85.6 (CH), 76.0 (CH), 72.0 (C), 46.8 (CH_2_), 37.8 (CH_2_) ppm. LC-MS(ES+) m/z 633.2 (M+H)^+ .^ HRMS (ES+) for C_36_H_32_N_4_O_7_^+^ (M+H)^+^ calcd 633.2344, found 633.2342.

The synthesis of compounds **2** and **4** is outline in **Scheme 3**. The starting diol was converted into a diamine by mesylation, displacement with sodium azide and hydrogenation. Monotritylation was achieved by the use of less than one equivalent of tritylchloride in the presence of trimethylamine using a 1/1 mixture of pyridine and DMF as solvent at room temperature. Compounds **2** and **4** were obtained by reaction of the primary amine with the corresponding isocyanate in THF at 40°C.

**Scheme 3:[18]** (i) MsCl (5eq), py, rt, 1.5h, yield: 80%; (ii) NaN_3_ (5 eq), DMF, 100°C, 1h, yield: 70%; (iii) H_2_, 10% Pd/C (10%w/w), MeOH, rt, 16 and then HCl/MeOH, yield: 90%; (iv) TrtCl (0.8eq), Et_3_N (3eq), py/DMF 1/1, rt, 36h, yield: 18%; (v) isocyanate, THF, 40^0^C.

**Ethyl 4-(3-(4-(2,4-dioxo-3,4-dihydropyrimin-1(*2H*)-yl)-2-((tritylamino)methyl)butyl)uredio)butanoate (Compound 2)**

¹H NMR (500 MHz, d-6 DMSO) **** 11.16 (s, 1H), 7.55 (d, 1H, *J*=7.8 Hz), 7.41 (d, 6H, *J*=7.5 Hz), 7.29-7.26 (m, 6H), 7.17 (dd, 3H, *J*=7.2 Hz), 5.82-5.79 (m, 1H), 5.72 (t, 1H, *J*=6.1 Hz), 5.51 (dd, 1H, *J*=2.0, 7.8 Hz), 4.08-4.03 (m, 2H), 3.67-3.52 (m, 2H), 3.24 - 3.18 (m, 1H), 3.12 - 3.05 (m, 1H), 3.01 - 2.88 (m, 4H), 2.29 - 2.23 (m, 3H), 1.96 - 1.81 (m, 2H), 1.64 - 1.55 (m, 4H), 1.47-1.41 (m, 1H), 1.20 - 1.16 (m, 3H) ppm; ^13^C-NMR (125 MHz, d-6 DMSO)  173.2 (C), 164.2 (C), 158.8 (C), 151.4 (C), 146.7 (C),  (CH),  (CH),  (CH), 126.4 (CH), 101.3 (CH), 70.9 (C), 60.2 (CH_2_), 46.1 (CH_2_), 44.8 (CH_2_), 37.8 (CH), 31.4 (CH_2_), 29.7 (CH_2_), 26.0 (CH_2_), 14.6 (CH_3_) ppm. LC-MS(ES+) m/z 612.3 (M+H)^+^ _._ HRMS (ES+) for C_35_H_42_N_5_O_5_^+^ (M+H)^+^ calcd 612.3186, found 612.3247.

**Ethyl 4-(3-(4-(2,4-dioxo-3,4-dihydropyrimin-1(*2H*)-yl)-2-((tritylamino)methyl)butyl)uredio)benzoate (Compound 4)**

¹H NMR (500 MHz, d-6 DMSO) **** 11.17 (s, 1H), 8.80 (s, 1H), 7.84 (d, 2H, *J*=8.9 Hz), 7.57 (d, 1H, *J*=7.9 Hz), 7.48 (d, 2H, *J*=8.7 Hz), 7.41 (d, 6H, *J*=7.3 Hz), 7.25-7.22 (m, 6H), 7.16-7.13 (m, 3H), 6.20 (t, 1H, *J*=6.0 Hz), 5.52 (dd, 1H, *J*=1.9, 7.9 Hz), 4.28 (q, 2H, *J*=7.1 Hz), 3.70 - 3.57 (m, 2H), 3.27 - 3.21 (1H, m), 2.82 - 2.77 (1H, m), 2.02 - 1.89 (m, 2H), 1.72 - 1.59 (m, 2H), 1.56 - 1.48 (m, 1H), 1.31 (t, 3H, *J*=7.1 Hz) ppm; ^13^C-NMR (125 MHz, d-6 DMSO)  166.0 (C), 164.2 (C), 155.5 (C), 151.4 (C), 146.6 (C), 146.0 (CH),  (C),  (CH),  (CH),  (CH), 126.4 (CH), 122.4 (C), 117.1 (CH), 101.4 (CH), 70.9 (C), 60.6 (CH_2_), 46.1 (CH_2_), 44.8 (CH_2_), 39.1 (CH_2_), 39.0 (CH_2_), 37.4 (CH), 19.8 (CH_2_), 14.7 (CH_3_) ppm. LC-MS(ES+) m/z 646.3 (M+H)^+^ _._ HRMS (ES+) for C_38_H_40_N_5_O_5_^+^ (M+H)^+^ calcd 646.3029, found 646.3059.

1. Nguyen, C., et al., *Deoxyuridine Triphosphate Nucleotidohydrolase as a Potential Antiparasitic Drug Target.* Journal of Medicinal Chemistry, 2005. **48**(19): p. 5942-5954.

2. Gilbert, I.N., Corinne; Ruda, Gian Filippo; Schhipani, Alessandro; Kasinathan, Ganasan; Johansson, Nils-Gunnar; Pacanowska, Dolores Gonzalez, *Preparation of pyrimidinediones as deoxyuridine triphosphate nucleotidohydrolase (dUTPase) inhibitors for treatment of parasitic infections. PCT int. Appl. (2005), WO 2005065689 A1 20050721.*
