# Supplementary figures and images for: Validation of Plasmodium falciparum dUTPase as the target of 5′-tritylated deoxyuridine analogues with anti-malarial activity
Source: Malar J. 2019 Dec 3;18:392. doi: 10.1186/s12936-019-3025-2 (PMC6889535; doi:10.1186/s12936-019-3025-2)

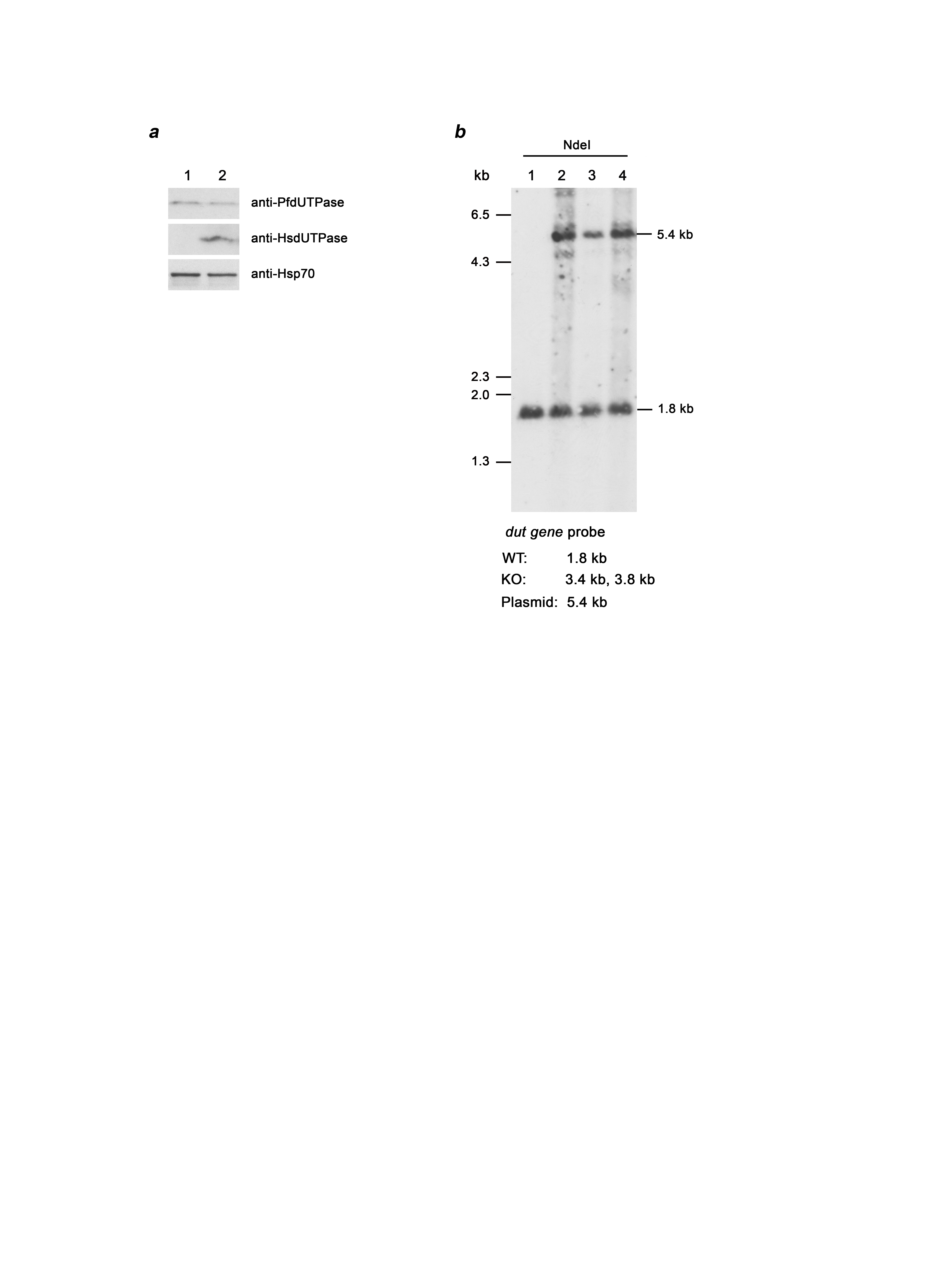

Supplement: Supplementary file 2 — Additional file 2. Attempt to disrupt the Pfdut gene in Plasmodium falciparum overexpressing HsdUTPase. a Overexpression of HsdUTPase in Plasmodium 3D7 cells cotransfected with pHrBl-Hsdut and pHH1-dutKO. Western blotting was performed with polyclonal antibodies raised against PfdUTPase and HsdUTPase. Hsp70 was used as loading control. Lane 1, extracts of non-transfected 3D7 cells; lane 2, extracts of cells cotransfected with pHrBl-Hsdut and pHH1-dutKO. b Southern blot analysis of the cell line cotransfected with pHrBl-Hsdut and pHH1-dutKO after one (lane 2), two (lane 3) and four (lane 4) cycles of drug pressure. Genomic DNA of the 3D7 parental line (lane 1) and the transfected cell line were digested with NdeI. The Southern blot was probed with a fragment of the Pfdut coding sequence. The 1.8 kb band corresponding to the endogenous locus is present in the four genomic DNAs, whereas the episome (5.4 kb) is present only in lanes 2, 3 and 4. Extra bands of 3.4 kb and 3.8 kb indicative of integration events were not detected. [file 12936_2019_3025_MOESM2_ESM.tif]
