# Supplementary material for: Validation of Plasmodium falciparum dUTPase as the target of 5′-tritylated deoxyuridine analogues with anti-malarial activity
Source: Malar J. 2019 Dec 3;18:392. doi: 10.1186/s12936-019-3025-2 (PMC6889535; doi:10.1186/s12936-019-3025-2)
Supplement: Supplementary file 3 — Additional file 3. Scheme depicting the role of dUTPase in pyrimidine metabolism. UMP/CMPK (putative), uridine monophosphate/cytidine monophosphate kinase; NDK, nucleoside-diphosphate kinase; CTPS, cytidine triphosphate synthetase; RNR, ribonucleotide reductase; dUTPase, deoxyuridine 5’-triphosphate nucleotido-hydrolase; DHFR-TS, dihydrofolate reductase / thymidylate synthase; TMPK, thymidine monophosphate kinase. [file 12936_2019_3025_MOESM3_ESM.pptx]

## Slide 1
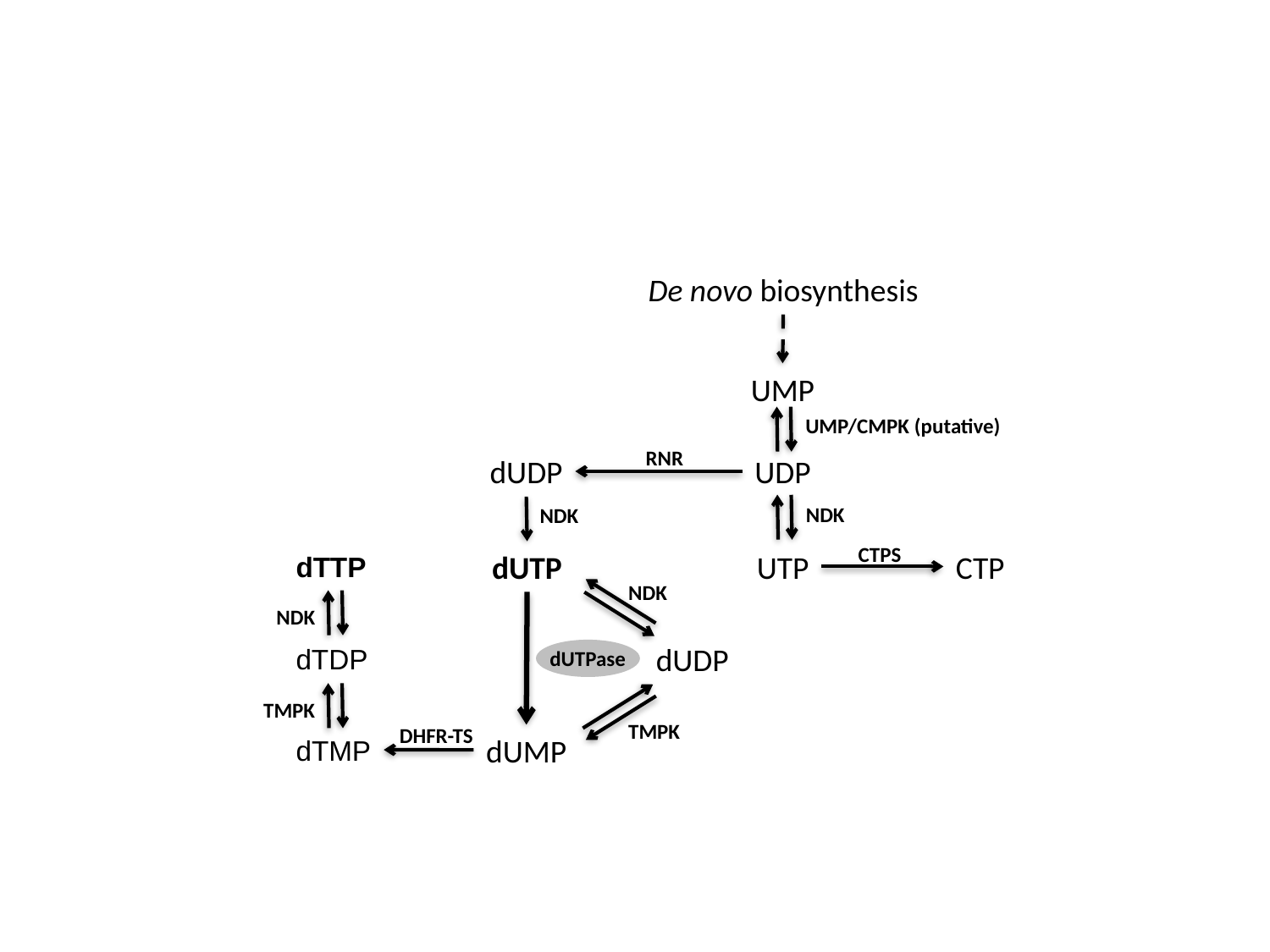

De novo biosynthesis
UMP
UMP/CMPK (putative)
RNR
dUDP
UDP
NDK
NDK
CTPS
dUTP
UTP
CTP
dTTP
NDK
NDK
dUDP
dTDP
dUTPase
TMPK
TMPK
DHFR-TS
dUMP
dTMP
